# Molecular Biology of the Cell, Sixth Edition; ISBN: 9780815344643; and Molecular Biology of the Cell, Sixth Edition, The Problems Book; ISBN 9780815344537

**DOI:** 10.3390/ijms161226074

**Published:** 2015-11-26

**Authors:** Stephen Bustin

**Affiliations:** Postgraduate Medical Institute, Anglia Ruskin University, Chelmsford CM1 1SQ, UK; stephen.bustin@anglia.ac.uk

## Abstract

The latest edition maintains the excellence and appeal of the previous editions. Its clear text and outstanding illustrations, together with the complementary problems book make this an essential companion for students and lecturers alike.

## What Is New?

- Diagrams, illustrations and photographs are either new or vastly improved, making them clear and easier to understand.
- A “What We Don’t Know” section challenges the reader to think outside the confines of the immediate topic discussed in the book.
- The end-of-chapter problems have been brought up-to-date and include all of the new topics introduced in this edition.
- The separate problems book is superb.

Writing and illustrating a text book on a topic as elaborate as the molecular biology of the cell is a huge undertaking, made all the more difficult by the continuous advances and unexpected revisions characteristic of this topic. Yes, there are some fundamental biological concepts that underpin this wide ranging subject, but an appreciation of the work being carried out in this field also requires an understanding of other more specialised subject areas such as biochemistry, biophysics, bioinformatics and biomechanics. The task of condensing all this information into a reasonably concise and readable book that retains its relevance for a number of years is daunting. Having to include appropriate illustrations that clarify and explain all this material makes such an undertaking a mammoth exercise. Furthermore, the competition from freely and instantly accessible online information requires any such book to offer a unique and cost-effective solution to the needs of impecunious (at least as far as study-associated expenses are concerned) students.

A perusal of the “Molecular Biology of the Cell” suggests that the authors have succeeded brilliantly in producing just such a work. The previous editions of this book have already established it as the definitive port of call for anyone interested in obtaining not just an overview but also an understanding of this topic. This sixth edition not only confirms the book’s pre-eminence but manages to extend it. The writing is very clear and the chapters progress as a narrative, making the experience not only interesting, but also managing to place everything into an appropriate context. Importantly, the information is also very much up to date: for example, the regulation of gene expression by noncoding RNAs is explained in detail, including the roles of small and long noncoding RNAs as well as the bacterial CRISPR system. I also particularly enjoyed the detailed information provided on the complicated issue of stem cells in the intestine in Chapter 22, together with wonderful illustrations and photographs. The breadth of information provided is vast: the first few chapters deal with basic biological and biochemical concepts, with the next few chapters describing genetic principles and regulatory aspects of gene expression. This is followed by extensive information about experimental techniques and new technologies used to study cell structure, analyse gene expression patterns and sequence genomes. For example, real-time PCR and Illumina as well as Ion Torrent sequencing methods are explained and even nanopore technology and quantum dots are mentioned, as are a wide range of methods used to analyse small molecules, proteins and cells. A new and incredibly useful addition is a section on “mathematical analysis of cell functions”. It demonstrates how mathematical analysis of the dynamics of molecular interactions such as those regulating gene expression can highlight the roles played by protein/promoter and protein/protein interaction as well as protein stability in generating or repressing transcriptional signals. The translation of the multiple and complex steps involved in these molecular communications into equations provides quantitative information useful for predicting cellular behaviour. Since many biologists are mathematically challenged, the clarity of presentation of these concepts will be very much appreciated. The book continues with detailed information on cellular organisation and structure as well as interaction of multicellular organisms and ends with descriptions of pathogens and the workings of the immune system. Each chapter ends with a list of essential references; a minor criticism is that it would have been useful to include Pubmed IDs, where relevant. Finally, there is an extensive glossary followed by an index.

The text is interspersed with succinct summaries, which serve to refocus the reader and there are numerous sections headed by “What We Don’t Know”, which poses questions that encourage the reader to not just take in all the information provided but inspire more detailed probing into that topic. A nicely illustrated “Problem” section at the end of each chapter also helps the reader deepen his/her understanding of the information acquired in that chapter. Figures enhance the text and structure/function relationships and interactions of proteins and cells are beautifully depicted. Photographs are often used to translate diagrams and schematics into the real world and, incidentally, help demonstrate how beautiful nature is at the sub-microscopic level.

I am especially impressed by the grouping of relevant information about any topic in panels, which convey in clear language and superb illustrations the basic knowledge required to understand that topic. The unique aspect of this way of presenting information is that it provides highly focused, yet comprehensive detail that is not readily available from a single source elsewhere, not even online. For example, one panel illustrates the basic chemistry responsible for giving biological molecules their characteristic properties, then provides information on thermodynamics and so makes it easier to follow the biochemical pathways that complete that panel.

The textbook is complemented by an equally comprehensive problems book, which “aims to help students appreciate the ways in which understanding of how cells work…can be further explored through experiments and simple calculations”. This aim is certainly achieved as nearly half the book (400 pages) is taken up with information, questions and exercises grouped into sections on “terms to learn”, “definitions”, “thought problems”, “calculations”, “data handling” and, something especially appreciated by my first-year medical student daughter, “medical links”. Again illustrations are superb and serve explain the background to the questions. Another 400 pages are taken up with detailed answers, again including illustrations where they clarify the subject matter. My only criticism is that the answers to the problems posed in the textbook are also answered here (over the last 150 pages), making it necessary to acquire both books. I think these answers should be provided at the end of the main book. I also very much like the fact that questions are not just answered, but that there are a large number of relevant references associated with these answers. This really encourages students to go back to the original source of whatever information is being discussed and start to learn how scientific questions are addressed in real life.

Whilst both books are probably targeted at advanced undergraduate and postgraduate students, I have found them invaluable as an accessible first and reliable source of information on topics I am less familiar with. Hence I am certain that it will be very useful, even essential, to anyone interested in molecular and cell biology, regardless of what stage in their career they are at. Lecturers in particular will love the emphasis on questions and exercises. In summary, the sixth edition of “Molecular Biology of the Cell”, is an exceptionally useful learning and teaching aid that provides clear, in-depth and up-to-date information on cellular molecular biology itself, techniques used to study this topic as well as complementary branches of science. Its complementary companion “The Problems Book” makes both books uniquely useful and important for student and teacher alike and together they are the most useful aids I can think of for anyone interested in learning about and understanding the molecular biology of the cell.

